# Commute trips in Norwegian cities: Data combining trip characteristics and revealed mode choice

**DOI:** 10.1016/j.dib.2021.107319

**Published:** 2021-08-22

**Authors:** Erik Bjørnson Lunke, Nils Fearnley, Jørgen Aarhaug

**Affiliations:** Institute of Transport Economics, Gaustadalleéen 21, Oslo 0349, Norway

**Keywords:** Public transport, Commuting, Revealed preference, Google maps, National travel survey

## Abstract

Most transport mode choice studies rely on subjective responses to hypothetical questions (stated preference), or on revealed preferences. In stated preference studies, trip characteristics are exact, but there is a range of sources of errors and biases in the responses. Revealed preference surveys suffer the opposite: The choice is exact (i.e. observed) but trip attributes are uncertain – and even more uncertain when it comes to transport modes not chosen. Our dataset goes a long way in solving these problems. The data set combines real travel behaviour and mode choice data from the Norwegian National Transport Survey (NTS) with trip characteristics collected from Google maps travel planner. From the NTS, we have extracted all commute trips conducted by either private car or public transport (PT) into ten major cities in Norway with exact origin and destination coordinates. The NTS data also comprises information about age, gender, household, income and car availability. From Google maps, we have extracted trip characteristics for these trips – for both the mode chosen and the mode not chosen. This data includes total travel time, the number of interchanges, wait time, walk time, and in-vehicle time. This data can be used to study how different trip characteristics influence the probability of choosing PT over private car on commute journeys.

## Specifications Table

**Table:** 

Subject	Social science
Specific subject area	Travel behaviour studies
Type of data	Table
How data were acquired	Transport mode use, trip origin and destination points and personal characteristics were gathered from the Norwegian National Transport Survey NTS (https://doi.org/10.18712/NSD-NSD2163-v11). Trip characteristics were extracted from Google Maps’ API^a^, using the origin and destination points from the NTS trips.
Data format	Raw
Parameters for data collection	The NTS was conducted among a representative sample of the Norwegian population over 13 years old in 2013 and 2014. The trip characteristics data were gathered using the origin and destination points of the NTS trips, calculating PT and car travel in the morning rush hour on a typical week day.
Description of data collection	The NTS data were collected through phone based interviews in 2013 and 2014. The trip characteristics data were collected by feeding the origin and destination coordinates of NTS trips in Google Maps’ API and extracting information on PT and car travel on these trip segments.
Data source location	Country: Norway Municipalities: Oslo, Fredrikstad, Drammen, Skien, Sandefjord, Arendal, Kristiansand, Stavanger, Bergen, Ålesund, Trondheim, Bodø and Tromsø. The data includes certain variables from the 2013/14 NTS. This primary data source is available from the Norwegian Centre for Research Data (NSD): https://doi.org/10.18712/NSD-NSD2163-v11.
Data accessibility	With the article
Related research article	E. B. Lunke, N. Fearnley, J. Aarhaug, Public transport competitiveness vs. the car: Impact of relative journey time and service attributes, Research in Transport Economics. https://doi.org/10.1016/j.retrec.2021.101098.

a Application programming interface.

## Value of the Data

• The data combines revealed commute mode choice with detailed information on travel time with car and public transport (PT), as well as other important PT trip characteristics, such as waiting times and number of transfers, for commute trips in urban areas in Norway. This combination is not often used in traditional travel behaviour research.
• Travel behaviour researchers can use these data to analyse and test established relationships between travel time, trip characteristics and mode choice on commute trips. This is highly relevant information for policymakers and other practitioners working to reduce the use of private car.
• The data can, among other possibilities, be used to further investigate how travel time, travel time disparities and the efficiency of the PT service influence the probability of commuters choosing PT over the private car.

## 1 Data Description

The data set consists of 5951 unique commuting trips conducted in the thirteen largest urban municipalities in Norway^1^ in 2013 and 2014. For each of these trips, we have extracted the transport mode used (either public transport (PT) or car), as well as the following personal characteristics of the commuters: Age, gender, education level and household income level. Secondly, the data set includes trip characteristics and geographical variables for each trip segment, extracted from the Google maps API. The trip characteristic variables include PT and car use (dummy variables), travel time with car, travel time with PT, travel time ratio (PT/car), number of transfers, over 500 m walking distance to nearest PT stop (dummy variable), more than 5 min waiting time during interchange (dummy variable), and finally a dummy variable defining if there are more than eight departures per hour on the selected PT service. All trip characteristics, for both car and PT, are extracted for all trips, regardless of the chosen transport mode. Geographic variables include one dummy variable defining if the trip is conducted in Oslo municipality (the largest city in Norway), and a dummy variable defining if the trip length is over 20 km.

The raw data set is available with this article. The descriptive characteristics of the data set can be found in Table 1 in Lunke et al. [1].

## 2 Experimental Design, Materials and Methods

The data set is a combination of a primary data source (the 2013/14 Norwegian National Transport Survey (NTS)) and the data we have collected ourselves from the Google Maps API. The first data source is the 2013/14 NTS, which is a nationwide survey consisting of around 60,000 interviews and nearly 200,000 registered trips [2]. From the NTS, we have extracted all commuting journeys (trips to work) conducted by either car or PT, ending in one of thirteen urban municipalities in Norway. Only trips conducted by commuters with a driving license and access to a private car were selected. This totals 5951 trips. In nine out of ten cases, the respondents have only conducted one such trip on the interview day. From the NTS, we extracted variables on mode choice (public transport (PT) or car), start and end point location (coordinates), as well as personal characteristics of the commuters.

Next, we have extracted travel characteristics for both car and PT from Google Maps’ journey planner for all the aforementioned commute trips. The extraction has been conducted by feeding Google's API with all journey start and end point coordinates. For simplicity, we selected the same arrival time for all trips, although the actual timing of the commute trips does vary. We have calculated trips that arrive at the end point at 08:30 am on a Wednesday in 2019, in order to get a representative time for commute travel. This arrival time is the most common among Norwegian commuters, according to the NTS. The difference in when data was collected between the NTS (2013/14) and the Google data (2019) poses a possible source or error. However, the infrastructure for car and PT travel in the study area has been quite stable over this time period, and we assume that in most cases there has been little change in the travel time of the studied trip segments.

From Google Maps’ API, we received a detailed data set containing information on all trip legs for each trip segment. A trip leg is defined by a specific transport mode. For example, if a commute trip by PT includes walking to a bus stop, travelling by bus to a new bus stop, walking to a train station, travelling by train to a new station and finally walking from the train station to the final destination, the trip includes five legs. For each leg, Google provided information on start and end time, transport mode (either walking or a specific PT mode), journey time and journey length. From this we derived the aforementioned trip characteristics. To define possible waiting during an interchange, we simply subtracted the journey time of a walk between stops from the time between the arrival of the first mode and the departure of the second mode. For car, we received the journey time (in real traffic during the selected time of travel) from the origin to the destination point.

## Ethics Statement

The survey data included in this data set comes from a primary data source. This data has been collected following the requirements of the Norwegian centre for research data (NSD), and the use of and distribution of the data is approved by NSD. The rest of our data set is collected from the Google maps journey planner which is openly available.

## CRediT Author Statement

**Erik B. Lunke**: Conceptualization, Methodology, Software, Validation, Formal analysis, Investigation, Resources, Data Curation, Writing – Original draft, Writing – Review & Editing, Visualization, Project administration, Funding acquisition; **Nils Fearnley:** Conceptualization, Methodology, Validation, Investigation, Resources, Writing – Original draft, Writing – Review & Editing, Visualization, Funding acquisition; **Jørgen Aarhaug:** Conceptualization, Methodology, Validation, Investigation, Writing – Original draft, Writing – Review & Editing, Visualization.

## Declaration of Competing Interest

The authors declare that they have no known competing financial interests or personal relationships which have or could be perceived to have influenced the work reported in this article.

## Appendix Supplementary materials

Image, application 1

## Footnotes

1 The thirteen municipalities are: Oslo, Fredrikstad, Drammen, Skien, Sandefjord, Arendal, Kristiansand, Stavanger, Bergen, Ålesund, Trondheim, Bodø and Tromsø

## References

[1] Lunke E.B., Fearnley N., Aarhaug J. (2021). Public transport competitiveness vs. the car: impact of relative journey time and service attributes. Res. Transp. Econ..

[2] Hjorthol R., Engebretsen Ø., Uteng T.P. (2014).

